# Cerebral Small Vessel Disease and Enlarged Perivascular Spaces-Data From Memory Clinic and Population-Based Settings

**DOI:** 10.3389/fneur.2019.00669

**Published:** 2019-06-25

**Authors:** Bibek Gyanwali, Henri Vrooman, Narayanaswamy Venketasubramanian, Tien Yin Wong, Ching-Yu Cheng, Christopher Chen, Saima Hilal

**Affiliations:** ^1^Memory Aging and Cognition Centre, National University Health System, Singapore, Singapore; ^2^Department of Pharmacology, National University of Singapore, Singapore, Singapore; ^3^Departments of Radiology and Medical Informatics, Erasmus University Medical Center, Rotterdam, Netherlands; ^4^Raffles Neuroscience Centre, Raffles Hospital, Singapore, Singapore; ^5^Singapore National Eye Center, Singapore Eye Research Institute, Singapore, Singapore; ^6^Departments of Epidemiology and Radiology and Nuclear Medicine, Erasmus University Medical Center, Rotterdam, Netherlands

**Keywords:** enlarged perivascular spaces, cerebral small vessel disease, memory clinic, population-based, magnetic resonance imaging

## Abstract

**Background:** Enlarged perivascular spaces (ePVS) are common finding on magnetic resonance imaging (MRI) in elderly. ePVS are thought to be associated with cerebral small vessel disease (SVD) such as white matter hyperintensities (WMH), lacunes, and cerebral microbleeds (CMBs). However, the different location of SVD and its relationship to ePVS distribution requires further investigation.

**Objective:** To study the association between location and severity of SVD with ePVS from memory clinic and population-based settings.

**Methods:** This study includes patients from an ongoing memory clinic based case-control study and participants from the population-based: Epidemiology of Dementia in Singapore study (EDIS). All participants underwent a comprehensive standardized evaluation including physical, medical and neuropsychological assessment and a brain MRI. CMBs and lacune location were categorized into strictly lobar, strictly deep and mixed, and ePVS location into centrum semiovale and basal ganglia. WMH volume was automatically segmented and was classified into anterior and posterior distribution. Negative binomial regression models were constructed to analyse associations between SVD and ePVS and the rate ratios (RR) and 95% confidence intervals (CI) were reported.

**Results:** Of 375 patients (median age = 73 years) from memory clinic and 583 participants (median age = 70 years) from EDIS, the median total ePVS count was 17.0 and 7.0, respectively. Increased severity of SVD was not associated with total ePVS counts in both memory clinic and EDIS study. Analysis with the location of SVD and ePVS also showed similar results. However, in EDIS study, presence of ≥2 lacunes [RR = 1.61, 95% CI = 1.3, 2.30, *p* = 0.009], presence of ≥2 CMBs [RR = 1.40, 95% CI = 1.08, 1.83, *p* = 0.012], and higher volume of WMH [RR = 1.41, 95% CI = 1.10, 1.81, *p* = 0.006] were associated with basal ganglia ePVS independent of age, gender and vascular risk factors.

**Conclusion:** In this study, we found that the ePVS were not associated with the location and severity of SVD in the memory-clinic patients. However, only severity of SVD was associated with basal ganglia ePVS in the population-based setting. Our findings will need to be studied further in different cohorts so as to understand the mechanism underlying different SVD types in subclinical and clinical phases as well as for predicting cognitive decline.

## Introduction

Cerebral small vessel disease (SVD) is considered as one of the leading causes of cognitive decline, physical disability and dementia in the elderly population (1, 2). SVD represents a group of pathological processes affecting the small arteries, arterioles, venules, and capillaries in the brain, resulting in various ischemic, hemorrhagic, and inflammatory damage (1, 3). White matter hyperintensities (WMH), lacunes, and cerebral microbleeds (CMBs) are MRI signatures of SVD and have been extensively studied in relation to cognition, clinical outcome and associations with other markers of SVD (4–6). In recent years, enlarged perivascular spaces (ePVS) have emerged as another feature of SVD, however their occurrence may also be non-pathogenic as they appear during normal aging (7).

Perivascular spaces are thought to be interstitial fluid-filled spaces surrounding the penetrating vessels in the brain. Physiologically, perivascular spaces are important for the drainage of interstitial fluid and regulating immune response (7). When enlarged, these perivascular spaces are commonly observed on MRI of elderly people (8). ePVS are mainly seen in the centrum semiovale and basal ganglia but may also appear in the hippocampus and the brain stem (9).

Previous studies have shown that ePVS are not only associated with other SVD markers such as WMH, lacunes, and CMBs (7, 10–12) but also with aging and vascular risk factors such as hypertension (13–16). By contrast, a few recent studies have shown that ePVS are not associated with WMH (16) and other cardiovascular risk factors (7). Furthermore, it is suggested that different location of ePVS may indicate different pathophysiological mechanisms such as cerebral amyloid angiopathy and hypertensive arteriopathy (7, 12). Previous studies have examined centrum semiovale and basal ganglia ePVS in relation to lobar and deep CMBs (12, 15). However, very few studies have taken into account the association between the location of lacunes and WMH with the location of ePVS (10, 17).

We aim to investigate the association between different locations and severity of SVD markers such as WMH, lacunes, and CMBs with different locations of ePVS (centrum semiovale and basal ganglia) in a spectrum of diseased patients (memory-clinic) to a population-based (Epidemiology of Dementia in Singapore study) setting.

## Materials and Methods

### Study Population

In this study, participants were recruited from two studies in Singapore. The first is a memory-clinic based case-control study, where patients (age ≥ 50 years) were recruited from memory clinics at the National University Hospital from August 2010 to December 2016. Cases were participants with subjective memory complains and impairment on neuropsychological assessment and were diagnosed with cognitive impairment no dementia (CIND) and dementia. CIND was defined as impairment in at least one cognitive domain on comprehensive neuropsychological test, but did not meet the criteria for dementia according to Diagnostic and Statistical Manual for Mental Disorder-Fourth Edition (DSM-IV). Dementia was diagnosed according to the DSM-IV criteria. The controls were individuals who had no objective cognitive impairment on comprehensive neuropsychological tests or any functional decline and were diagnosed as No Cognitive Impairment (NCI).

Of the total 501 patients from the memory clinics, 18 had incomplete or poor quality scan, 12 did not undergo MRI scans (3 were claustrophobic, 1 refused, 2 were uncooperative or could not follow instructions, and 6 had contraindications for MRI), and 96 had no ePVS grading (because the primary sequences required for ePVS grading i.e., T1 and T2-weighted images were missing and/or had motion artifacts which restricted our grading), leaving a final sample of 375 cases for analysis.

The second is the Epidemiology of Dementia in Singapore study (EDIS), which recruited multi-ethnic (Chinese, Indian, and Malay) participants from the Singapore Epidemiology of Eye Disease (SEED) study (18). SEED is a large population based study among Chinese [Singapore Chinese Eye Study (SCES)], Malay [Singapore Malay Eye Study (SiMES-2)], and Indians [Singapore Indian Eye Study (SINDI-2)]. Participants from the SEED study who were ≥60 years and were screened-positive on the Abbreviated Mental Test (AMT) or self-reported progressive forgetfulness (PF) were invited to participate in the EDIS study from August 2010 to July 2015 (19). EDIS study also used similar diagnostic criteria as memory clinic, where participants were diagnosed as NCI (individuals who had no objective cognitive impairment on comprehensive neuropsychological tests or any functional decline), CIND (individuals with impairment in at least one cognitive domain on comprehensive neuropsychological test, but did not meet the criteria for dementia according to DSM-IV), and Dementia (diagnosed according to the DSM- IV criteria).

A total of 300 Chinese and 323 Malay screen-positive participants agreed to take part in the second phase of this study, which included an extensive neuropsychological test battery and brain MRI. The present analysis was restricted to Chinese and Malay as the ePVS data was only available in these two ethnicities. Of the 623 participants, 36 had no MRI scans and 4 had poor quality MRI scans, leaving 583 (284 Chinese and 299 Malays) cases for the final analysis.

The memory clinic study was approved by the National Healthcare Group Domain-Specific Review Board. For the EDIS study, ethics approval was obtained from both the Singapore Eye Research Institute and National Healthcare Group Domain-Specific Review Board. This study is conducted in accordance with the Declaration of Helsinki. A written informed consent was obtained from all participants or their caregivers prior to the recruitment for this study.

### Demographics and Vascular Risk Factors

All participants were administered a detailed questionnaire to collect information on age, gender, years of formal education, and smoking history. Previous medical history of hypertension, diabetes, and hyperlipidemia was noted and subsequently verified by medical records. Hypertension was defined as systolic blood pressure ≥140 mmHg and/or diastolic blood pressure ≥90 mmHg during the examination, or previous diagnosis of hypertension, or the use of antihypertensive medications. Hyperlipidemia was defined as total cholesterol level ≥4.14 mmol/l during the examination or previous diagnosis of hyperlipidemia, or the use of lipid-lowering medications. Diabetes mellitus was defined as glycated hemoglobin ≥6.5% during the examination, or previous diagnosis of diabetes mellitus, or the use of glucose-lowering medications.

### Neuroimaging

MRI scans of all the participants from both EDIS study and the memory clinics were performed at the Clinical Imaging Research Centre of the National University of Singapore, using a 3T Siemens Magnetom Trio Tim Scanner system with a 32-channel head coil. The standardized neuroimaging protocol in this study included a three dimensional T1-weighted sequence, a T2-weighted sequence, fluid-attenuated inversion recovery (FLAIR), and a susceptibility weighted image (SWI). Quantitative MRI analyses were performed using automated segmentation procedures at the Department of Medical Informatics, Erasmus University Medical Center, the Netherlands, using a model-based methodology (FreeSurfer, v.5.1.0) on T1 weighted images (TR = 7.2 ms, TE = 3.3 ms, matrix = 256 × 256 × 180 mm^3^). For each participant, the following MRI-based markers were analyzed:

- CMBs were graded on SWI sequence according to the Brain Observer Micro Bleed Scale (20). CMBs were manually classified in both lobes (left and right) into two different locations: lobar (cortex/gray-white junction, subcortical white matter) and deep (basal ganglia, thalamus, internal and external capsule, brainstem, and cerebellum). CMBs were then further divided into three groups: strictly lobar CMBs (presence of CMBs exclusively in lobar region), strictly deep CMBs (presence of CMBs exclusively in deep region), and mixed CMBs (CMBs distributed in both lobar and deep locations). The total number of CMBs in each location was recorded and calculated as the sum of strictly lobar, strictly deep, and mixed CMBs. Total CMBs was further categorized into three groups according to CMBs burden: 0 CMB, presence of 1 CMB, and presence of ≥2 CMBs (21).
- Lacunes were defined as round or ovoid lesions involving the subcortical regions, 3–15 mm in diameter, with a low signal on T1-weighted images and FLAIR, a high signal on T2-weighted images and a hyperintense rim with a center following the cerebrospinal fluid intensity (22). Similarly, lacunes were classified into two different locations: lobar (when located in frontal, parietal, temporal, occipital, insula, and centrum semiovale) and deep (when located in basal ganglia, thalamus, internal, and external capsule) (23). Lacunes were further divided into three groups: strictly lobar lacunes (presence of lacunes exclusively in lobar region), strictly deep lacunes (presence of lacunes exclusively in deep region), and mixed lacunes (presence of lacunes distributed in both lobar and deep locations). The total number of lacunes in each location was recorded and was calculated as sum of strictly lobar, strictly deep, and mixed lacunes. Total lacunes were further categorized into three groups according to lacunes burden: 0 lacune, presence of 1 lacune, and presence of ≥2 lacunes.
- ePVS were defined as round or linear hypointense lesions on T1 weighted and hyperintense lesion on T2 weighted images. When lesion is ≥1 mm, it is considered as dilated. In this study, ePVS were visually counted in four different regions of the brain: centrum semiovale, basal ganglia, mesencephalon, and hippocampus. Centrum semiovale EPVS were graded in the slice 10 mm above the lateral ventricle, whereas basal ganglia EPVS were graded to the level of the anterior commissure. ePVS in mesencephalon and hippocampus, were graded in all slices (24). Total ePVS was calculated as sum of centrum semiovale, basal ganglia, hippocampus, and mesencephalon ePVS. Due to the small number of ePVS in mesencephalon and hippocampus, we did not use these regions in further analysis.
- WMH volume was quantified using T1 and T2 weighted images. The image preprocessing steps and the tissue classification algorithm have been described elsewhere (25). Briefly, a k-nearest-neighbor (kNN) classifier technique was used to classify voxels into cerebrospinal fluid (CSF), gray matter, normal white matter and WMH. Volumes (ml) were calculated for all biomarkers from these segmentations. Region-specific WMH volume was calculated for frontal, parietal, occipital, and temporal lobes. Total WMH volume was calculated as the sum of WMH volumes in the above mentioned five regions. In this study, frontal WMH volume was classified as anterior and sum of parietal and occipital WMH volume as posterior (26–28). Total WMH volume was further categorized into tertiles to represent severity of WMH.

Intra-rater agreement for lacunes, CMBs, and ePVS was good to excellent, which has been published previously (8, 29).

### Statistical Analysis

CMBs and lacunes were treated as counts and categorical variables. For categorical data, we classify CMB and lacunes as: presence vs. absence and 1 vs. 0, ≥2 vs. 0, and by location (strictly lobar vs. no, strictly deep vs. no, and mixed vs. no).WMH volumes were logarithmically transformed due to skewed distribution and were divided into tertiles (second tertile vs. first tertile and third tertile vs. first tertile). We chose to present the results with ePVS as count variable in this study because the numbers of participants with no ePVS were too few in binary category. SVD markers were treated as determinants and ePVS as outcomes. In our secondary analysis, we divided our study subjects into two groups i.e., NCI group which included NCI and cognitive impairment group which included CIND and Dementia. In order to analyze the association between location and severity of SVD markers with ePVS counts, negative binomial regression was constructed with rate ratios (RR) and 95% confidence intervals (CI). All models were adjusted for age, gender, hypertension, hyperlipidemia, and diabetes. Results were considered significant at *p* < 0.05. In view of multiple testing performed between SVD and ePVS, we used Bonferroni correction to obtain revised statistical significance level of 0.05/2 ~ 0.025. All the data were analyzed using SPSS software package (version 25).

## Results

The characteristics of the study population from the memory clinic and EDIS study is shown in Table 1. Patients from memory clinic were older. EDIS participants had burden of hypertension where as participants from memory clinic had higher SVD burden. The median number (interquartile range) of total ePVS in memory clinic was 17.0 (11.0) and in EDIS, 7.0 (8.0).

**Table 1:** Characteristics of study participants.

**Baseline variables**	**Memory clinic (*n* = 375)**	**EDIS (*n* = 583)**
**Demographic factors**		
Age, median (IQR)	73 (12)	70 (11)
Gender (Female), *n* (%)	199 (53.1)	319 (54.7)
**Cardiovascular determinants**		
Hypertension, *n* (%)	264 (70.4)	485 (83.2)
Hyperlipidemia, *n* (%)	273 (72.8)	412 (70.7)
Diabetes, *n* (%)	132 (35.2)	176 (30.2)
**MRI markers**		
Presence of Lacunes, *n* (%)	106 (28.3)	106 (18.2)
Strictly lobar lacunes, *n* (%)	32 (8.5)	50 (8.6)
Strictly deep lacunes, *n* (%)	43 (11.5)	27 (4.6)
Mixed lacunes, *n* (%)	31 (8.3)	29 (5.0)
Presence of CMBs, *n* (%)	156 (41.6)	215 (36.5)
Strictly lobar CMBs, *n* (%)	81 (21.6)	110 (18.9)
Strictly deep CMBs, *n* (%)	30 (8.0)	32 (5.5)
Mixed CMBs, *n* (%)	45 (12.0)	73 (12.5)
Total WMH volume, ml, median (IQR)	3.5 (11.0)	2.1 (6.0)
Anterior WMH volume, ml, median (IQR)	0.7 (3.3)	0.3 (1.4)
Posterior WMH volume, ml, median (IQR)	0.5 (3.1)	0.3 (1.5)
Total ePVS, median (IQR)	17.0 (11)	7.0 (8.0)
Centrum semiovale ePVS, median (IQR)	10.0 (9.0)	3.0 (5)
Basal ganglia ePVS, median (IQR)	3.0 (3)	2.0 (3)

*SD, standard deviation; IQR, inter quartile range; WMH, white matter hyperintensity; ePVS, enlarged perivascular spaces; EDIS, Epidemiology of Dementia in Singapore*.

The association between severity of SVD with total and region-specific ePVS in the memory clinic population is shown in Table 2. Increased severity of CMBs, lacunes and WMH was not associated with increased number of ePVS. Region-specific analysis also showed that increased severity of SVD was not associated with centrum semiovale ePVS and basal ganglia ePVS. Similarly, stratifying CMBs and lacunes into 0, 1, and ≥2 and WMH volume into tertiles did not change the results.

**Table 2:** Association between severity of SVD and ePVS (Memory clinic).

**SVD markers**	**Total ePVS RR (95% CI)**	**Centrum semiovale ePVS RR (95% CI)**	**Basal ganglia ePVS RR (95% CI)**
Total CMBs	1.00 (0.99, 1.01)	1.00 (0.98, 1.01)	1.01 (0.99, 1.02)
1 CMB	0.96 (0.71, 1.30)	1.00 (0.74, 1.36)	0.96 (0.69, 1.33)
≥2 CMBs	1.06 (0.83, 1.36)	1.02 (0.80, 1.31)	1.04 (0.79, 1.37)
Total lacunes	1.04 (0.93, 1.16)	0.98 (0.87, 1.01)	1.10 (0.98, 1.25)
1 lacune	1.13 (0.84, 1.51)	1.13 (0.84, 1.52)	0.98 (0.71, 1.36)
≥2 lacunes	1.17 (0.76, 1.49)	0.88 (0.62, 1.24)	1.37 (0.96, 1.97)
Total WMH volume	1.00 (0.94, 1.00)	1.00 (0.99, 1.00)	1.00 (0.99, 1.00)
Second tertile	1.04 (0.80, 1.35)	1.06 (0.82, 1.39)	0.96 (0.72, 1.28)
Third tertile	1.09 (0.83, 1.43)	1.07 (0.82, 1.41)	1.02 (0.75, 1.37)

*RR, rate ratio; CI, confidence interval; CMBs, cerebral microbleeds; ePVS, enlarged perivascular spaces; WMH, white matter hyperintensity. All values adjusted for age, gender, hypertension, hyperlipidemia, and diabetes*.

The association between locations of SVD with ePVS in memory clinic population is shown in Table 3. Increased number of lobar, deep, and mixed CMBs as well as lacunes and higher volumes of anterior and posterior WMH were not associated with ePVS (total ePVS, centrum semiovale ePVS, and basal ganglia ePVS). When treating CMBs and lacunes as categorical data (presence vs. absence) in regression analysis, no association was again observed (Supplementary Table 1).

**Table 3:** Association between location of SVD and ePVS (Memory clinic).

**SVD markers**	**Total ePVS RR (95% CI)**	**Centrum semiovale ePVS RR (95% CI)**	**Basal ganglia ePVS RR (95% CI)**
Strictly lobar CMBs	1.00 (0.98, 1.02)	0.99 (0.97, 1.01)	1.01 (0.99, 1.03)
Strictly deep CMBs	1.01 (0.88, 1.15)	0.99 (0.86, 1.12)	1.02 (0.87, 1.20)
Mixed CMBs	1.00 (0.99, 1.02)	1.00 (0.98, 1.01)	1.01 (0.99, 1.02)
Strictly lobar lacunes	0.99 (0.76, 1.29)	0.93 (0.71, 1.23)	1.03 (0.78, 1.35)
Strictly deep lacunes	1.00 (0.79, 1.27)	0.98 (0.77, 1.25)	0.92 (0.71, 1.18)
Mixed lacunes	1.05 (0.92, 1.19)	1.00 (0.88, 1.14)	1.15 (1.00, 1.32)
Anterior WMH volume	1.00 (0.98, 1.01)	0.99 (0.98, 1.01)	1.00 (0.98, 1.02)
Posterior WMH volume	1.00 (0.99, 1.01)	1.00 (0.99, 1.01)	1.00 (0.99, 1.01)

*RR, rate ratio; CI, confidence interval; CMBs, cerebral microbleeds; ePVS, enlarged perivascular spaces; WMH, white matter hyperintensity. All values adjusted for age, gender, hypertension, hyperlipidemia, and diabetes*.

The association between severity of SVD with ePVS in EDIS participants is shown in Table 4. Increased numbers of CMBs, lacunes, and increased severity of WMH volume were not associated with increased number of ePVS. Furthermore, region-specific analysis of ePVS as centrum semiovale ePVS and basal ganglia ePVS also did not show any significant association with SVD. However, on stratifying CMBs and lacunes into 1 vs. 0 and ≥2 vs. 0; presence of ≥2 lacunes [RR = 1.61, 95% CI = 1.3, 2.30, p = 0.009] and presence of ≥2 CMBs [RR = 1.40, 95% CI = 1.08, 1.83, p = 0.012] were associated with basal ganglia ePVS. Higher volume of WMH in third tertile was associated with basal ganglia ePVS [RR = 1.41, 95% CI = 1.10, 1.81, p = 0.006]. Moreover, presence of ≥2 lacunes was found to be associated with reduced centrum semiovale ePVS counts [RR = 0.61, 95% CI = 0.42, 0.88, p = 0.009]. These associations survived multiple testing.

**Table 4:** Association between severity of SVD and ePVS (EDIS).

**SVD markers**	**Total ePVS RR (95% CI)**	**Centrum semiovale ePVS RR (95% CI)**	**Basal ganglia ePVS RR (95% CI)**
Total CMBs^*^	1.00 (0.99, 1.01)	0.99 (0.98, 1.00)	1.00 (0.99, 1.01)
1 CMB	1.04 (0.84, 1.30)	0.93 (0.73, 1.18)	1.09 (0.85, 1.40)
≥2 CMBs	1.18 (0.93, 1.51)	1.00 (0.78, 1.29)	1.40 (1.08, 1.83)^*^
Total lacunes	1.07 (0.93, 1.22)	0.89 (0.76, 1.04)	1.21 (0.70, 1.92)
1 lacune	1.33 (1.00, 1.76)	1.43 (1.06, 1.92)	1.14 (1.00, 1.48)
≥2 lacunes	1.05 (0.75, 1.47)	0.61 (0.42, 0.88)^*^	1.61 (1.13, 2.30)^*^
Total WMH volume	1.00 (0.99, 1.12)	1.00 (0.99, 1.00)	1.00 (0.99, 1.00)
Second tertile	0.97 (0.78, 1.20)	0.90 (0.72, 1.14)	1.15 (0.90, 1.47)
Third tertile	1.07 (0.85, 1.34)	0.81 (0.63, 1.03)	1.41 (1.10, 1.81)^*^

*RR, rate ratio; CI, confidence interval; CMBs, cerebral microbleeds; ePVS, enlarged perivascular spaces; WMH, white matter hyperintensity; EDIS, Epidemiology of Dementia in Singapore. All values adjusted for age, gender, hypertension, hyperlipidemia, and diabetes. Bold values represents statistically significant associations at p < 0.05*.

* Statistically significant after Bonferroni correction (0.05/2 ~ 0.025).

The association between locations of SVD with ePVS in EDIS participants is shown in Table 5. Location-specific analysis of SVD did not show any significant association with total ePVS counts. On repeating this analysis with region-specific ePVS counts, increased number of lobar, deep and mixed CMBs, and lacunes were not associated with centrum semiovale ePVS. However, there was borderline association between increased number of strictly lobar lacunes [RR = 1.27, 95% CI = 1.00, 1.62, p = 0.055] and mixed lacunes [RR = 1.16, 95% CI = 0.99, 1.36, p = 0.070] with basal ganglia ePVS. Higher anterior and posterior WMH volumes were not associated with centrum semiovale ePVS or with basal ganglia ePVS.

**Table 5:** Association between location of SVD and ePVS (EDIS study).

**SVD markers**	**Total ePVS RR (95% CI)**	**Centrum semiovale ePVS RR (95% CI)**	**Basal ganglia ePVS RR (95% CI)**
Total CMBs	1.00 (0.99, 1.01)	0.99 (0.98, 1.00)	1.00 (0.99, 1.01)
Strictly lobar CMBs	0.98 (0.83, 1.16)	0.92 (0.78, 1.10)	1.10 (0.92, 1.32)
Strictly deep CMBs	1.22 (0.84, 1.77)	1.27 (0.86, 1.88)	0.88 (0.57, 1.35)
Mixed CMBs	1.00 (0.99, 1.01)	0.99 (0.98, 1.00)	1.00 (0.99, 1.01)
Strictly lobar lacunes	1.12 (0.88, 1.42)	1.04 (0.80, 1.36)	1.27 (1.00, 1.62)
Strictly deep lacunes	1.23 (0.86, 1.75)	1.21 (0.83, 1.77)	1.25 (0.85, 1.83)
Mixed lacunes	1.00 (0.86, 1.16)	0.78 (0.63, 0.94)	1.16 (0.99, 1.36)
Anterior WMH volume	1.00 (0.99, 1.01)	1.00 (0.99, 1.01)	1.01 (0.99, 1.02)
Posterior WMH volume	1.00 (0.99, 1.01)	1.00 (0.99, 1.01)	1.00 (0.99, 1.01)

*RR, rate ratio; CI, confidence interval; CMBs, cerebral microbleeds; ePVS, enlarged perivascular spaces; WMH, white matter hyperintensity; EDIS, Epidemiology of Dementia in Singapore. All values adjusted for age, gender, hypertension, hyperlipidemia, and diabetes*.

In EDIS participants, when treating CMBs and lacunes as categorical data (presence vs. absence), no significant association was again observed between presence of SVD and ePVS. However, there was border line association between presence of CMBs [RR = 1.23, 95% CI = 0.99, 1.51, *p* = 0.058] and lacunes [RR = 1.29, 95% CI = 0.97, 1.91, *p* = 0.056] with basal ganglia ePVS. Region-specific analysis of CMBs and lacunes showed border line association between mixed CMBs [RR = 1.18, 95% CI = 0.94, 1.09, *p* = 0.057], and mixed lacunes [RR = 1.36, 95% CI = 0.74, 2.27, *p* = 0.054] with basal ganglia ePVS (Supplementary Table 2).

On performing secondary analysis among cognitive impairment and NCI groups, we found location and severity of SVD markers were not associated with ePVS counts in memory clinic and population-based setting. However, in EDIS study, presence of ≥2 lacunes [RR = 1.59, 95% CI = 1.09, 2.31, *p* = 0.015], ≥2 CMBs [RR = 1.40, 95% CI = 1.08, 1.98, *p* = 0.014], and higher volume of WMH in third tertile [RR = 1.45, 95% CI = 1.08, 1.94, *p* = 0.012] were associated with basal ganglia ePVS in cognitively impaired group only. Moreover, among individuals with NCI in EDIS study, increased number of strictly lobar lacunes [RR = 0.37, 95% CI = 0.17, 0.77, *p* = 0.008] and higher WMH volume in third tertile [RR = 0.48, 95% CI = 0.29, 0.78, *p* = 0.003], more specifically anterior [RR = 0.07, 95% CI = 0.66, 0.90, *p* = 0.001] and posterior WMH volume [RR = 0.76, 95% CI = 0.66, 0.88, *p* < 0.001] were associated with lower centrum semiovale ePVS counts [data not shown].

## Discussion

This study demonstrated that increased severity of SVD markers were not associated with total ePVS counts in memory clinic and population-based settings. Region specific analysis did not change these results. However, in EDIS study presence of ≥2 lacunes, ≥2 CMBs, and higher volume of WMH were associated with basal ganglia ePVS independent of age, gender, and vascular risk factors. We did not find an association between location of SVD with ePVS in centrum semiovale and basal ganglia.

Till date, this is the first study to examine the association between location and severity of SVD with total and region specific ePVS counts. Previous studies have shown that lobar CMBs were associated with centrum semiovale ePVS and deep CMBs with basal ganglia ePVS (12, 15). Furthermore, higher WMH volume was associated with basal ganglia ePVS (12). However, in this study we found that total and region specific ePVS counts were not associated with location of SVD in both memory clinic and population-based settings. This might be due to the fact that first, ePVS are considered as an early MRI feature in the spectrum of SVD markers (16). Previous studies have shown that the WMH, CMBs, ePVS, and lacunes co-occur suggesting a common pathophysiological mechanism underlying these lesions (30). Second, during the different stages of cognitive impairment, other factors such as inflammation, amyloid, tau may attenuate the effect the traditional cardiovascular risk factors such as hypertension, hyperlipidemia, and diabetes (31, 32). Third, cerebral amyloid angiopathy and hypertensive arteriopathy may be due to two separate mechanisms in preclinical stages, but at the advanced stage, they co-occur and manifest as mixed pathology (30). Since the participants of the memory clinic have a higher burden of cardiovascular risk factors, we speculate that they might already be in more advanced stage of cerebrovascular damage and hence a ceiling effect is likely to be observed (8), which may explain the lack of association between location and severity of SVD with ePVS. Moreover, there might be differences in study population (hospital/memory clinic vs. population-based), different MRI modalities i.e., field strength (1.5T vs. 3T vs. 7 T), methods in ePVS grading (whole brain vs. particular slice vs. particular hemisphere), sequences used and variation in risk factor profile as well as the inclusion criteria of the study population. It has been shown that, perivascular spaces are present in abundance throughout the healthy brain (7) and when enlarged can be visualized on MRI (8). It is possible that ePVS differ from other markers of SVD and may represent a non-pathological process of aging with no clinical consequences.

The prevalence of SVD has been suggested to differ among ethnicities due to differences in vascular risk factors, genetic, and environmental susceptibility (29). It is reported that the burden of SVD is higher in Asians compared to Caucasians due to higher prevalence of cardiovascular risk factors (33). Hence, we speculate that Asians with higher SVD burden would have higher number of ePVS compared to Caucasians. By contrast, the ePVS counts in both memory clinics and EDIS study were lower than previous studies (7, 10, 17, 34, 35). This difference may be attributed to different grading method for ePVS. Similar results were also mentioned in a recent meta-analysis which included EDIS and other community-based samples from Austria, Hong Kong, Netherlands, and Germany. Despite using a harmonized method to grade ePVS in each study, Asians had lower number of ePVS, especially lowest in Hong Kong (mean ePVS = 2.1) compared to other cohorts in that study [Austria; mean ePVS = 30.8, Netherlands; mean ePVS = 16.7, Germany; mean ePVS = 8.2] (8).

Previous neuropathological studies have shown co-occurrence of ePVS with other SVD markers such as CMBs, WMH, and lacunes. However, it is difficult to conclude the chronological order of the occurrence of these markers as post-mortem samples are obtained at later stage of life when there is significant SVD burden. It is not clear that ePVS precede, follow or co-occur together with other SVD. We cannot exclude the possibility that ePVS of ≥1 mm may be non-pathogenic and may not have any significant effect on SVD. It has been shown that large perivascular spaces (≥3 mm in diameter) were associated with MRI markers of SVD and increased risk of dementia (36). We postulate that smaller ePVS are non-pathological and hence are not associated with SVD in this study.

Interestingly, in EDIS study, we found that the presence of ≥2 lacunes, CMBs, and higher WMH volume were associated with basal ganglia ePVS. We only found this association in population-based setting but not in memory-clinic study. Although no direct conclusion can be drawn from this study with respect to the association between ePVS and SVD, these results should be interpreted with caution. Notably, EDIS participants were relatively younger and had lower burden of vascular risk factors especially lower diabetes and hyperlipidemia hence, they may be at early stage of disease, whereas memory clinic participants were older and had higher burden of vascular risk factors and are at later stage of cognitive impairment. Furthermore, EDIS participants had a higher burden of hypertension compared to memory clinic subjects, which might have influenced the association however, previous studies on association between vascular risk factors and ePVS are controversial. It has been shown ePVS were associated with vascular risk factors such as hypertension (13–16) but a recent study did not report such findings (7). Hence, this finding leads us to further confirm that ePVS may be early marker of SVD. However, these results should be interpreted with caution. Interestingly in EDIS study, we found that the presence of ≥2 lacunes was associated with lower ePVS counts in centrum semiovale. In subgroup analysis, among the individuals with NCI in EDIS study increased number of strictly lobar lacunes was associated with lower ePVS count in centrum semiovale. The possible reason might be, due to similar MRI characteristics of ePVS and lacunes, where several ePVS may have been mis-graded as lacunes or vice versa and thus we might have underestimated the exact numbers of ePVS (22, 37–39). Similarly, in subjects with NCI in EDIS study, higher WMH volume were associated with lower counts of centrum semiovale ePVS. This might be explained by the fact that extensive WMH obscures ePVS grading especially in centrum semiovale and hence under estimates PVS counting in that region.

Our study has some limitations. First, as this is a cross-sectional study, we were unable to determine the cause-effect relationship and also the chronological order of SVD and temporal change of SVD over time. Second, even though we adjusted for several risk factors such as age, gender, hypertension, diabetes, and hyperlipidemia, we cannot ignore the possibility of residual confounding. Third, ePVS were graded at one particular slice for basal ganglia and centrum semiovale, we may have missed other possible ePVS. However, this method has shown strong correlation with whole brain grading approach (9). Fourth, as this study was conducted in Asian population, our results are not generalizable to other ethnicities. Fifth, the data on ≥3 mm ePVS is only present in EDIS study. There were only 25 subjects with ePVS ≥3 mm in this study. With this small number of cases, we were unable to find any association. Hence we are unable to perform analysis comparing the association of ePVS and SVD in small ePVS (≥1 mm) and large ePVS (≥3 mm). Strength of this study include, use of 2 different population i.e., memory clinic and population-based with different risk factor profile and SVD burden. All MRIs were done on 3T scanner and SVD markers were graded blinded to clinical history following similar protocol.

Our findings demonstrated that ePVS counts were not associated with markers of SVD in memory clinic and population-based settings in Asia. However, in population-based setting presence of ≥2 lacunes, ≥2 CMBs, and higher WMH volume were associated with basal ganglia ePVS. Our results need to be studied further in large cohorts with cross-sectional and longitudinal designs using consistent and harmonized methods for ePVS so as to understand the underlying mechanism of different SVD markers in subclinical and clinical phases as well as for predicting cognitive decline.

## Data Availability

All datasets generated for this study are included in the manuscript and/or the Supplementary Files.

## Ethics Statement

The memory clinic study was approved by the National Healthcare Group Domain-Specific Review Board. For the EDIS study, ethics approval was obtained from both the Singapore Eye Research Institute and National Healthcare Group Domain-Specific Review Board. This study is conducted in accordance with the Declaration of Helsinki. A written informed consent was obtained from all participants or their caregivers prior to the recruitment for this study.

## Author Contributions

BG participated in data acquisition, performed analysis, drafted, and revised manuscript. HV participated in MRI segmentation and revised manuscript. NV was responsible for provided intellectual advice and revised manuscript. TW and C-YC revised the manuscript. CC and SH were responsible for study design and concept, obtaining funding, overall supervision, and revised the manuscript.

### Conflict of Interest Statement

The authors declare that the research was conducted in the absence of any commercial or financial relationships that could be construed as a potential conflict of interest.
