# Supplementary material for: Cerebral Small Vessel Disease and Enlarged Perivascular Spaces-Data From Memory Clinic and Population-Based Settings
Source: Front Neurol. 2019 Jun 25;10:669. doi: 10.3389/fneur.2019.00669 (PMC6603207; doi:10.3389/fneur.2019.00669)
Supplement: Supplementary file 1 [file Table_1.docx]

**Supplementary Table 1: Association between location of SVD and ePVS (Memory clinic)**

| **SVD markers** | **Total ePVS**  **RR (95% CI)** | **Centrum semiovale ePVS**  **RR (95% CI)** | **Basal ganglia ePVS**  **RR (95% CI)** |
| --- | --- | --- | --- |
| Presence of CMBs | 1.10 (0.86, 1.40) | 1.03 (0.80, 1.31) | 1.14 (0.88, 1.50) |
| Presence of strictly Lobar CMBs | 1.08 (0.83, 1.41) | 1.13 (0.87, 1.48) | 0.98 (0.74, 1.31) |
| Presence of strictly Deep CMBs | 0.89 (0.60, 1.33) | 0.84 (0.56, 1.25) | 1.08 (0.70, 1.66) |
| Presence of Mixed CMBs | 1.01 (0.72, 1.41) | 0.92 (0.65, 1.29) | 1.01 (0.70, 1.46) |
| Presence of lacunes | 1.03 (0.83, 1.27) | 1.01 (0.82, 1.26) | 1.01 (0.80, 1.28) |
| Presence of strictly Lobar lacunes | 1.07 (0.72, 1.59) | 1.01 (0.68, 1.50) | 1.07 (0.69, 1.65) |
| Presence of strictly Deep lacunes | 1.06 (0.76, 1.49) | 1.03 (0.74, 1.45) | 0.93 (0.64, 1.35) |
| Presence of Mixed lacunes | 1.19 (0.81, 1.75) | 1.03 (0.69, 1.53) | 1.53 (1.00, 2.32) |

Abbreviations: RR, rate ratio; CI, confidence interval; CMBs, cerebral microbleeds; ePVS, enlarged perivascular spaces; WMH, white matter hyperintensity

All values adjusted for age, gender, hypertension, hyperlipidemia and diabetes.

**Supplementary Table 2: Association between location of SVD and ePVS (EDIS study)**

| **SVD markers** | **Total ePVS**  **RR (95% CI)** | **Centrum semiovale ePVS**  **RR (95% CI)** | **Basal ganglia ePVS**  **RR (95% CI)** |
| --- | --- | --- | --- |
| Presence of CMBs | 1.11 (0.93, 1.33) | 0.97 (0.79, 1.18) | 1.23 (0.99, 1.51) |
| Presence of strictly Lobar CMBs | 1.00 (0.80, 1.26) | 0.87 (0.68, 1.12) | 1.19 (0.92, 1.53) |
| Presence of strictly Deep CMBs | 1.26 (0.86, 1.85) | 1.24 (0.83, 1.84) | 0.98 (0.63, 1.51) |
| Presence of Mixed CMBs | 1.22 (0.93, 1.59) | 1.00 (0.75, 1.33) | 1.18 (0.94, 1.09) |
| Presence of lacunes | 1.21 (0.96, 1.53) | 1.09 (0.85, 1.39) | 1.29 (0.97, 1.91) |
| Presence of strictly Lobar lacunes | 1.25 (0.91, 1.73) | 1.23 (0.88, 1.72) | 1.10 (0.88, 1.83) |
| Presence of strictly Deep lacunes | 1.32 (0.88, 1.98) | 1.30 (0.85, 1.99) | 1.42 (0.91, 2.22) |
| Presence of Mixed lacunes | 1.06 (0.71, 1.59) | 0.67 (0.43, 1.04) | 1.36 (0.74, 2.27) |

Abbreviations: RR, rate ratio; CI, confidence interval; CMBs, cerebral microbleeds; ePVS: enlarged perivascular spaces; WMH, white matter hyperintensity; EDIS, Epidemiology of Dementia in Singapore

All values adjusted for age, gender, hypertension, hyperlipidemia and diabetes.
